# Adipose Tissue-Derived Mesenchymal Stem Cell Modulates the Immune Response of Allergic Rhinitis in a Rat Model

**DOI:** 10.3390/ijms20040873

**Published:** 2019-02-18

**Authors:** Nesrine Ebrahim, Yasser Mohammad Hassan Mandour, Ayman Samir Farid, Ebtesam Nafie, Amira Zaky Mohamed, Miriam Safwat, Radwa Taha, Dina Sabry, Safwa M. Sorour, Ahmed Refae

**Affiliations:** 1 Department of Histology and Cell Biology, Benha University, Benha, Qalyubia 13518, Egypt; nesrien.salem@fmed.bu.edu.eg; 2 Stem Cell Unit, Benha University, Benha, Qalyubia 13518, Egypt; 3 Department of Otorhinolaryngology, Faculty of Medicine, Benha University, Benha, Qalyubia 13518, Egypt; yasser.mandour@fmed.bu.edu.eg (Y.M.H.M.); ahmed.alrefaai@fmed.bu.edu.eg (A.R.); 4 Department of Clinical Pathology, Faculty of Veterinary Medicine, Benha University, Moshtohor, Toukh, Qalyubia 13736, Egypt; 5 Zoology Department, Faculty of Science, Benha University, Benha 13518, Egypt; ebtesam.nafe@fsc.bu.edu.eg; 6 Department of Microbiology, Faculty of Science, Tanta University, Tanta 31527, Egypt; a.shenawy.32016@alexu.edu.eg; 7 Department of Medical Biochemistry and Molecular Biology, Faculty of Medicine, Cairo University, Cairo 11562, Egypt; Miriam.safwat@kaseralainy.edu.eg (M.S.); radwa.taha@kaseralainy.edu.eg (R.T.); 8 Department of Medical Biochemistry, Faculty of Medicine, Cairo University, Cairo 11562, Egypt; dinasabry@kasralainy.edu.eg; 9 Molecular Biology and Stem Cell Unit, Faculty of Medicine, Cairo University, Cairo 11562, Egypt; 10 Department of Clinical Pharmacology, Faculty of Medicine, Benha University, Benha, Qalyubia 13518, Egypt; safwa.sorour@fmed.bu.edu.eg

**Keywords:** allergic rhinitis, antileukotriene, CCL11, immunoglobulins, mesenchymal stem cells, Montelukast, VCAM-1

## Abstract

This study was designed to investigate the potential effects and underlying mechanism of adipose tissue-derived mesenchymal stem cells (MSCs) on allergic inflammation compared to Montelukast as an antileukotriene drug in a rat model of allergic rhinitis (AR). The effect of MSCs was evaluated in albino rats that were randomly divided into four (control, AR, AR + Montelukast, and AR + MSCs) groups. Rats of AR group were sensitized by ovalbumin (OVA) and then challenged with daily nasal drops of OVA diluted in sterile physiological saline (50 μL/nostril, 100 mg/mL, 10% OVA) from day 15 to day 21 of treatment with/without Montelukast (1 h before each challenge) or MSCs I/P injection (1 × 10^6^ MCSs; weekly for three constitutive weeks). Both Montelukast and MSCs treatment started from day 15 of the experiment. At the end of the 5th week, blood samples were collected from all rats for immunological assays, histological, and molecular biology examinations. Both oral Montelukast and intraperitoneal injection of MSCs significantly reduced allergic symptoms and OVA-specific immunoglobulin E (IgE), IgG1, IgG2a and histamine as well as increasing prostaglandin E2 (PGE2). Further analysis revealed that induction of nasal innate cytokines, such as interleukin (IL)-4 and TNF-α; and chemokines, such as CCL11 and vascular cell adhesion molecule-1 (VCAM-1), were suppressed; and transforming growth factor-β (TGF-β) was up-regulated in Montelukast and MSCs-treated groups with superior effect to MSCs, which explained their underlying mechanism. In addition, the adipose tissue-derived MSCs-treated group had more restoring effects on nasal mucosa structure demonstrated by electron microscopical examination.

## 1. Introduction

Mesenchymal stem cells (MSCs) are multipotent cells that are capable of differentiating into three types of mesenchymal cells: adipocytes, osteoblasts, and chondrocytes [1]. MSCs represent an important stem cell population with multipotent capabilities that may have high utility for translational clinical applications [2]. MSC-based cell therapy has been demonstrated as a promising strategy in the treatment of immune diseases [3,4]. Although the therapeutic/immunomodulatory mode of action of MSCs is largely unknown [4], they exhibit a strong immunomodulation potential via their interaction with T lymphocytes, B lymphocytes, natural killer (NK) cells, and dendritic cells (DC) [5,6]. While the ability of MSCs to modulate immune systems has led to increasing interest in using MSCs as a promising therapeutic modality for allergic airway diseases, only few studies have demonstrated that MSCs can ameliorate allergic airway inflammatory diseases, including asthma [7] and AR [8,9,10]. Moreover, the immunomodulatory mechanisms of MSCs in allergic airway disease are only partly understood.

Allergic rhinitis (AR) is a chronic inflammatory disorder of the nasal airways, characterized by an imbalance of T-helper type 1/T-helper type 2 (T_H_1/T_H_2) cells and deficiency of regulatory T (Treg) cells [11]. The disease impairs the quality of life [12] and is associated with decreased learning, performance, and productivity at work and school and its economic impact is substantial [13]. AR is a common health problem in both children and adults, and nearly 20–30% of the world population suffers from this disease [14,15]. However, rhinitis is still underdiagnosed and not correctly treated [13].

The extreme stimulation of T_H_2 cells is thought to play a major role in initiating clinical features that include allergic sensitization and provoking airway hyperactivity [16]. Recent studies have suggested that T_H_2 cells and mast cells play crucial roles in the pathogenesis and process of allergic reactions during AR [17]. T_H_2 cells mediate the activation and maintenance of the allergens and secrete a population of T_H_2 cytokines, such as interleukin (IL)-4 and IL-13, as well as promoting antigen-specific immunoglobulin E (IgE) production by B cells [18,19]. IgE antibodies “sensitize” a group of cells including mast cells triggering degranulation of their vesicles and releasing a cascade of inflammatory mediators (histamine) causing localized inflammation, which produces an early phase of AR [20], while the late phase is characterized by increased numbers of T_H_2 lymphocytes, eosinophils, basophils, and neutrophils, which release cytokines and other mediator molecules such as leukotrienes [21]. 

Leukotrienes enhance inflammatory cells recruitment and activation, fibrosis, and airway remodeling (increasing the deposition of collagen below the basement membrane, enhancing collagen synthesis and degradation by fibroblasts). During the early phase of immune response, cysteinyl leukotrienes (CysLTs) are released by mast cells and basophils, while in the late phase they are synthesized by eosinophils and macrophages [22]. Antileukotriene drugs are classified, based upon their mechanism of action, into two groups. The first group is cysteinyl leukotriene receptor antagonists, which work by blocking the leukotriene receptor, and thus block the end organ response of leukotriene. This group includes Zafirlukast, Pranlukast, and Montelukast. Among CysLT1 receptor antagonists, Montelukast is the only drug approved for treatment of allergic rhinitis. The second group is leukotriene synthesis inhibitors (5-lipoxygenase inhibitor), which work by blocking the biosynthesis of cysteinyl leukotrienes and leukotriene B4 (LTB4) [23]. 

Current chemical drugs towards AR are limited to antihistaminic, local steroid, and antileukotriene drugs as monotherapy or combination therapy but with side-effects of long-term usage; thus, researchers have been trying to find different therapeutic approaches [24,25]. Therefore, the aim of this study was to compare the immunomodulatory effects of both MSCs and leukotriene antagonist in experimentally induced AR in a rat model.

## 2. Results

### 2.1. MSCs Identification in Culture and Tissue Tracking

**Figure 1:**
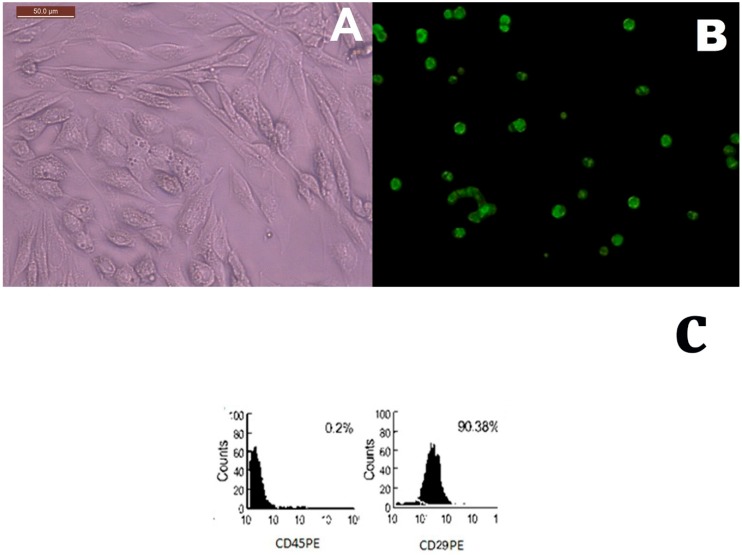
(**A**) An inverted microscope micrograph on day 14 from primary culture of mesenchymal stem cells showing many spindle-shaped stem cells, ×200. (**B**) Fluorescent microscopic image of tissue culture demonstrating the green fluorescence of MSCs labeled with GFP two weeks after implantation (fluorescent technique, ×200). (**C**) Flow cytometric analysis of surface antigens of MSCs, MSCs were positive for CD29 and negative for CD45.

The MSCs were identified after two weeks in culture with an inverted microscope as spindle-shaped adhesive cells (Figure 1A). MSCs labeled with GFP were observed in tissue culture using a fluorescent microscope (Figure 1B). Analysis of cell surface markers was performed to confirm mesenchymal stem cells preparations by CD29^+^ positive cells (90.38%) and CD45^−^ negative cells (0.20%) specific to MSCs (Figure 1C). 

### 2.2. Symptom Score

**Figure 2:**
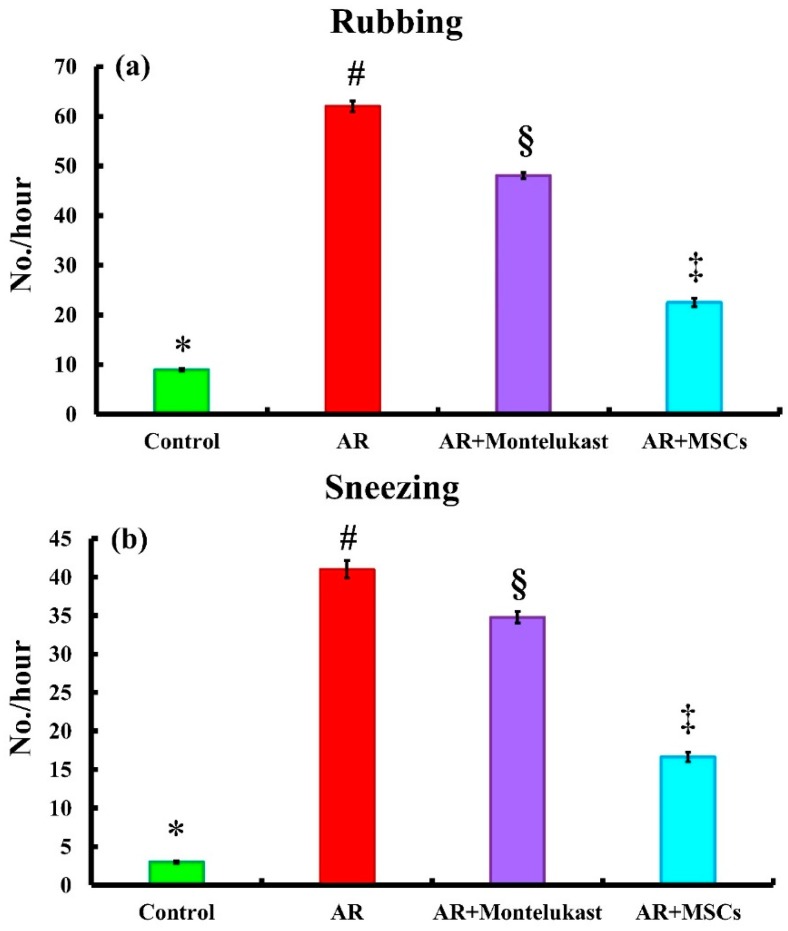
Systemic administration of MSCs reduced allergic symptoms. Rubbing (**a**) and sneezing (**b**) in different experimental groups. Different superscripts (*, #, §, and ‡) indicate significant differences among the experimental groups at *p* < 0.05. Data are shown as mean ± S.E.M, *n* = 6.

To investigate the role of MSCs in AR, we used an AR rat model and administered OVA with aluminum hydroxide gel for 15 days. We measured the number of sneezing and nose rubbing motions in one hour after challenging the rats with OVA via nasal inhalation. The results showed that the rats in the AR model group sneezed (41.00 ± 1.13 No./h) and rubbed (61.00 ± 1.11 No./h) their noses significantly) *p* < 0.05), more frequently than those in the control group (3.00 ± 0.16 and 8.95 ± 0.31 No./h, respectively). Interestingly, the sneezing and nasal rubbing numbers were significantly (*p* < 0.05) lower in the rats treated with multiple dosages of MCSs (16.63 ± 0.60 and 22.48 ± 0.84 No./h; respectively) from the commencement of OVA administration (Figure 2a,b) compared to AR model and (AR + Montelukast) groups. Simultaneously, we observed that the sneezing and rubbing numbers of the AR + Montelukast rats (34.87 ± 0.74 and 48.06 ± 0.58 No./h; respectively) showed a similar change after treatments with Montelukast and MSCs strategies. Notably, treatment with MSCs inhibits sneezing and rubbing frequencies more significantly than montelukast) *p* < 0.05). This result suggests that MSCs have a therapeutic effect on acute AR rats.

### 2.3. Biochemical Results

**Figure 3:**
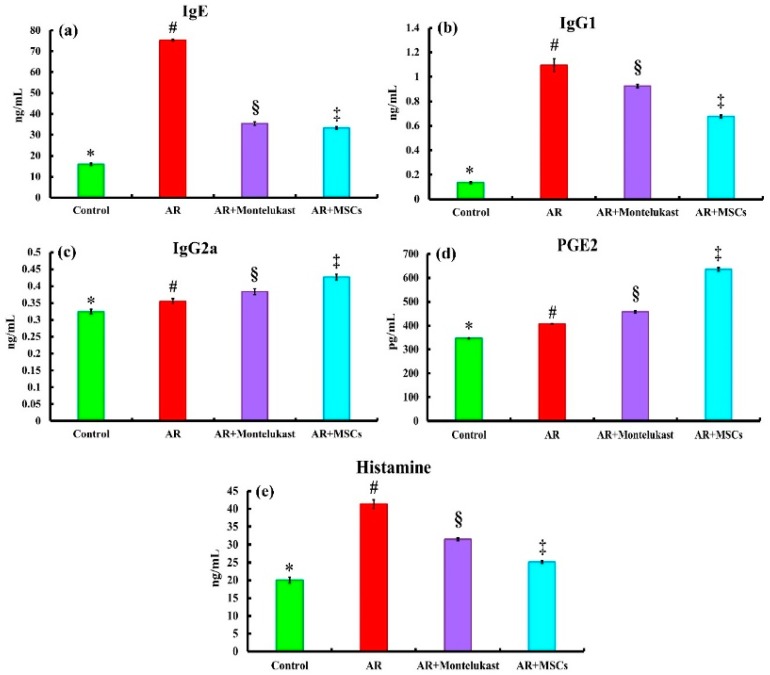
Systemic administration of MSCs decreases the serum levels of antigen-specific-antibody responses. There are significant decreases in OVA-specific IgE (**a**) IgG1 (**b**) and IgG_2_a (**c**), as well as increases in PEG2 (**d**) and histamine (**e**) levels in the sera of rats following the different treatments. Different superscripts (*, #, §, and ‡) indicate significant differences among the experimental groups at *p* < 0.05. Data are shown as mean ± S.E.M, *n* = 5–6.

To elucidate the mechanism underlying the therapeutic effects of Montelukast and MSCs on AR, we examined the production of OVA-specific IgE, IgG1, IgG2a, PGE2, and histamine by enzyme-linked immunosorbent assay (ELISA) (Figure 3). OVA-specific IgE, IgG1, and IgG2a levels were significantly (*p* < 0.05) higher in the AR group (Group II) (75.26 ± 0.50, 1.09 ± 0.05 and 0.35 ± 0.00 ng/mL; respectively) compared to the control group (Group I) (15.95 ± 0.59, 0.13 ± 0.00 and 0.32 ± 0.00 ng/mL; respectively). In the AR + Montelukast group (Group III), there were significant (*p* < 0.05) decreases in OVA-specific IgE (35.4 ± 0.84 ng/mL) and IgG2a (0.38 ± 0.00 ng/mL) compared to AR group (Group II). However, the AR+MSCs group (Group IV) showed significant (*p* < 0.05) decreases in OVA-specific IgE (33.35 ± 0.57 ng/mL), IgG1 (0.675 ± 0.01 ng/mL) and IgG2a (0.42 ± 0.00 ng/mL) compared to the AR group (Group II). 

Prostaglandin E2 (PGE2) is an eicosanoid lipid mediator that significantly participates in the pathogenesis of many inflammatory reactions. The PGE2 level was significantly (*p* <0.05) increased in groups AR (II) (406.50 ± 1.47 ng/mL), AR+Montelukast (III) (457.66 ± 4.53 ng/mL) and AR+MSCs (IV) (635.16 ± 7.95 ng/mL) compared to the control group (I) (346.70 ± 1.47 ng/mL). Interestingly, the magnitude of PGE2 elevation in MSCs-treated groups was significantly (*p* <0.05) higher than the AR and AR + Montelukast groups. 

Histamine is considered one of the mediators involved in local inflammatory response due to mast cell degranulation. Histamine levels were significantly (*p* < 0.05) increased in AR (II) (41.33 ± 1.14 ng/mL), AR + Montelukast (III) (31.48 ± 0.34 ng/mL) and AR + MSCs (IV) (25.13 ± 0.29 ng/mL) compared to the control group (I) (20.00 ± 0.81 ng/mL), while its level was significantly (*p* < 0.05) decreased in the MSCs-treated groups when compared with the AR and AR + Montelukast groups. It is worth mentioning that examination of all subgroups of the control group showed similar results regarding biochemical examinations; therefore, the results of subgroup Ia were used to represent this group.

### 2.4. Gene Expression Results of IL-4, TNF-α, TGF-β, VCAM-1 and CCL11 Genes in All Experimental Groups

**Figure 4:**
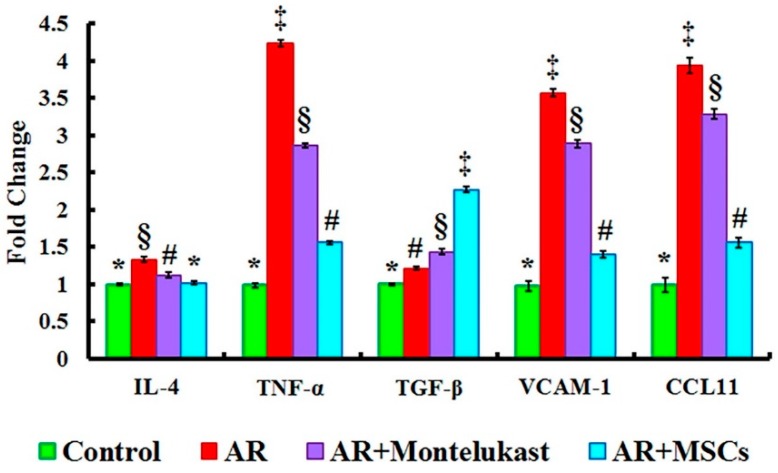
Quantitative analysis for relative IL-4, TNF-α, TGF-β, VCAM-1 and CCL11 genes expression after the indicated treatment with Montelukast or MSCs. Different superscripts (*, #, §, and ‡) at the same check point to significant differences at *p* < 0.05. Data are shown as mean ± S.E.M, *n* = 5.

To parallelly evaluate the molecular mechanisms underlying the immunomodulatory properties of MSCs, the mRNA expression of nasal cytokine was investigated (Figure 4). Gene expressions of IL-4, TNF-α, TGF- β, VCAM-1 and CCL11 were significantly up-regulated in the AR group (Group II) (1.33 ± 0.03, 4.23 ± 0.04, 1.21 ± 0.01, 3.57 ± 0.05 and 3.94 ± 0.10 fold change; respectively) compared to the control group (Group I) (0.99 ± 0.01, 0.98 ± 0.02, 1.00 ± 0.01, 0.97 ± 0.06 and 0.98 ± 0.09 fold change; respectively). However, IL-4, TNF-α, VCAM-1 and CCL11 genes were significantly down-regulated in AR+Montelukast group (Group III) (1.12 ± 0.04, 2.86 ± 0.03, 2.89 ± 0.05 and 3.28 ± 0.06 fold change; respectively) and AR+MSCs group (group IV) (1.01 ± 0.02, 1.55 ± 0.02, 1.40 ± 0.04 and 1.55 ± 0.07 fold change; respectively) groups when compared with the AR group (Group II), while the gene expression of TGF-β was significantly increased in both groups (Groups III [1.43 ± 0.04 fold change] and IV [2.27 ± 0.04 fold change]) compared to the AR group (Group II) (1.21 ± 0.01 fold change). Importantly, the magnitude of IL-4, TNF-α, TGF-β, VCAM1, and CCL11 genes expression variations in the AR+MSCs group was significant when compared with the AR + Montelukast group. 

### 2.5. Histological Results

Histological examination of the different subgroups of group I (control group) showed similar results; therefore, the results of subgroup Ia were used to represent this group.

#### 2.5.1. Hematoxylin and Eosin

**Figure 5:**
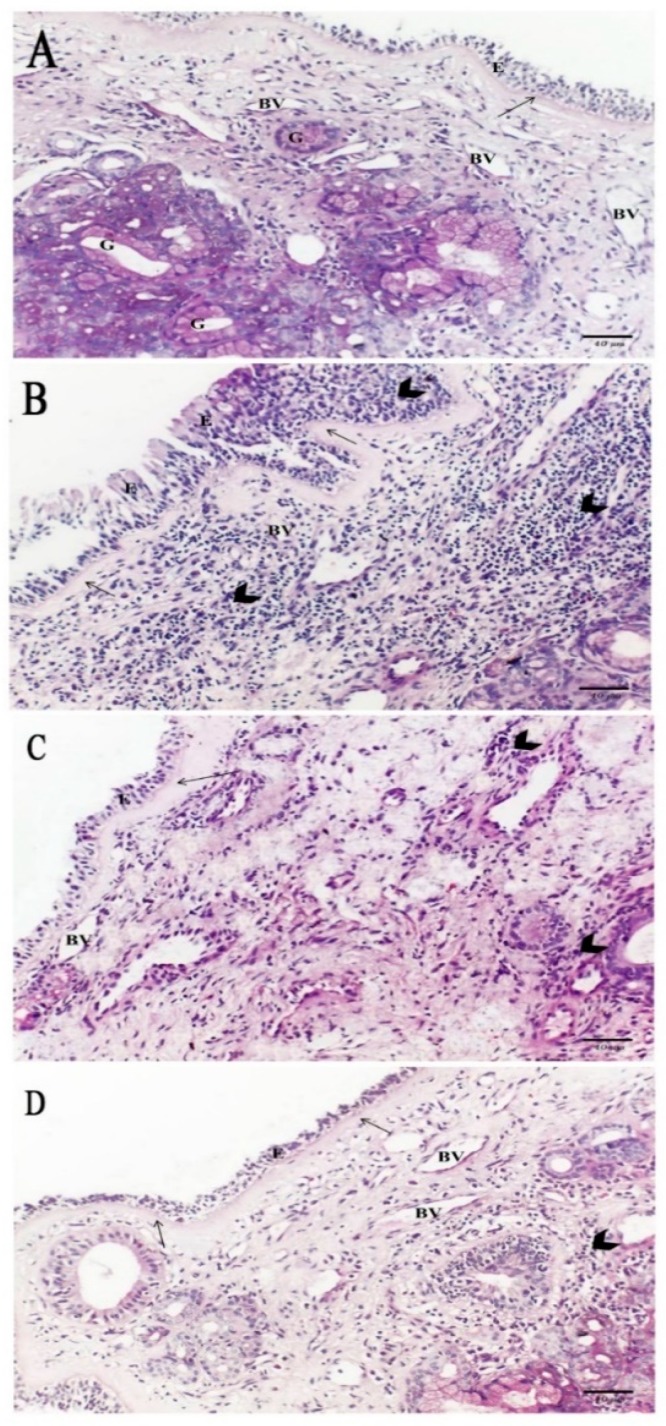
(**A**) A photomicrograph of a section in the nasal mucosa of group I (control group) displaying normal nasal mucosa with respiratory epithelium (E) resting on thin basal lamina (arrow) and lamina propria of loose connective tissue having multiple blood vessels (BV) and seromucinous glands (G). (**B**) A photomicrograph of a section in the nasal mucosa of AR group (Group II) showing impairment of respiratory epithelium (E) resting on apparently thick basal lamina (arrow) and lamina propria with infiltration of abundant cells (arrow head) and with few blood vessels (BV). (**C**) A photomicrograph of a section in the nasal mucosa of Group III displaying partial damage of respiratory epithelium (E) resting on apparently thick basal lamina (arrow) and lamina propria with infiltration of a few cells (arrow head) and with few blood vessels (BV). (**D**) A photomicrograph of a section in the nasal mucosa of Group IV displaying near normal nasal mucosa with overcrowded respiratory epithelium (E) resting on apparently thin basal lamina (arrow) and lamina propria of loose connective tissue having infiltration of a few cells (arrow head) and having many blood vessels (BV) [H&E × 200].

Sections of control group (Group I) revealed normal nasal cavity mucosa with respiratory epithelium (pseudostratified columnar ciliated with goblet cells). The cells were tall columnar cells with some invaginations mostly to increase surface area resting on thin basal lamina and lamina propria of loose connective tissue containing multiple blood vessels and seromucinous glands. Branching seromucinous glands were noticed, reflecting high secretory functions of these glands (Figure 5A). The AR group (Group II) showed damaged respiratory epithelium (the epithelial cells lost their positions, mucosa exfoliation) with a focal area of epithelial hypertrophy resting on apparently thick basal lamina and lamina propria with infiltration of numerous cells (lymphocytes, eosinophils and plasma cells) and few blood vessels with seromucinous glands (Figure 5B). In the AR+Montelukast group (Group III), there was partial damage of respiratory epithelium resting on thick basal lamina, and the lamina propria had infiltration of a few inflammatory cells with few blood vessels (Figure 5C). On the other hand, the AR + MSCs group (Group IV) showed normal nasal mucosa with overcrowded respiratory epithelium resting on thin basal lamina and lamina propria of loose connective tissue with infiltration of a few cells and many blood vessels with seromucinous glands (Figure 5D).

#### 2.5.2. Masson’s Trichrome

**Figure 6:**
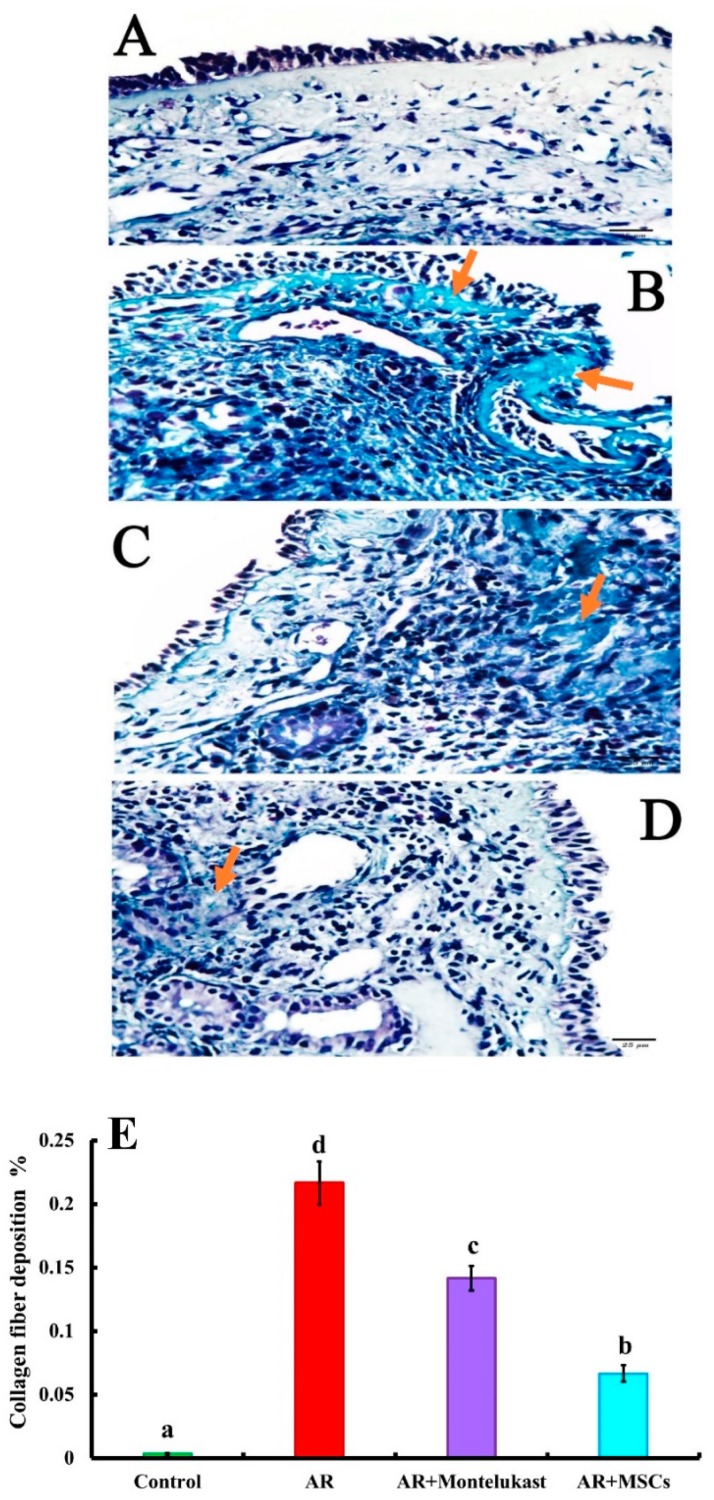
(**A**) A photomicrograph of a section in the nasal mucosa of the control group displaying minimal collagen fibers in lamina propria. (**B**) A photomicrograph of a section in the nasal mucosa of Group II displaying extensive accumulation of collagen fibers at the basal lamina and lamina propria (yellow arrow). (**C**) A photomicrograph of a section in the nasal mucosa of group III displaying accumulation of collagen fibers (yellow arrow) at the basal lamina and lamina propria. (**D**) A photomicrograph of a section in the nasal mucosa of Group IV displaying minimal collagen fibers in the lamina propria [Masson’s trichrome, ×400]. (**E**) A box plot showed the mean area percentage of collagen fibers in all experimental groups. Different superscripts (a, b, c, and d) indicate significant differences among the experimental groups at *p* < 0.05. Data are shown as mean ± S.E.M., *n* = 4.

Masson’s trichrome stained sections of the control group (Group I) demonstrated faint and minimal collagen fibers in the lamina propria of the nasal mucosa (Figure 6A). The AR group (Group II) showed marked accumulation of collagen fibers at the basal lamina and lamina propria (Figure 6B). In the AR + Montelukast group (Group III), there were accumulations of collagen fibers at basal lamina and lamina propria was minimally decreased (Figure 6C), while the AR+MSCs group (Group IV) showed minimal collagen fibers in the lamina propria (Figure 6D). The mean area percentage of collagen fibers accumulation for all groups was presented in Figure 6E. There were significant (*p* < 0.05) increases in the accumulation of collagen fibers in all groups that suffered from AR (Groups II, III and IV) (0.21 ± 0.01, 0.14 ± 0.00, and 0.06 ± 0.00 %; respectively) when compared with the control group (Group I) (00.00 ± 0.00 %). Meanwhile, the deposition of collagen fibers was significantly decreased in the group treated with MSCs (AR + MSCs group) when compared with the AR and AR + Montelukast groups.

### 2.6. The Transmission Electron Microscopy (T.E.M) Results

**Figure 7:**
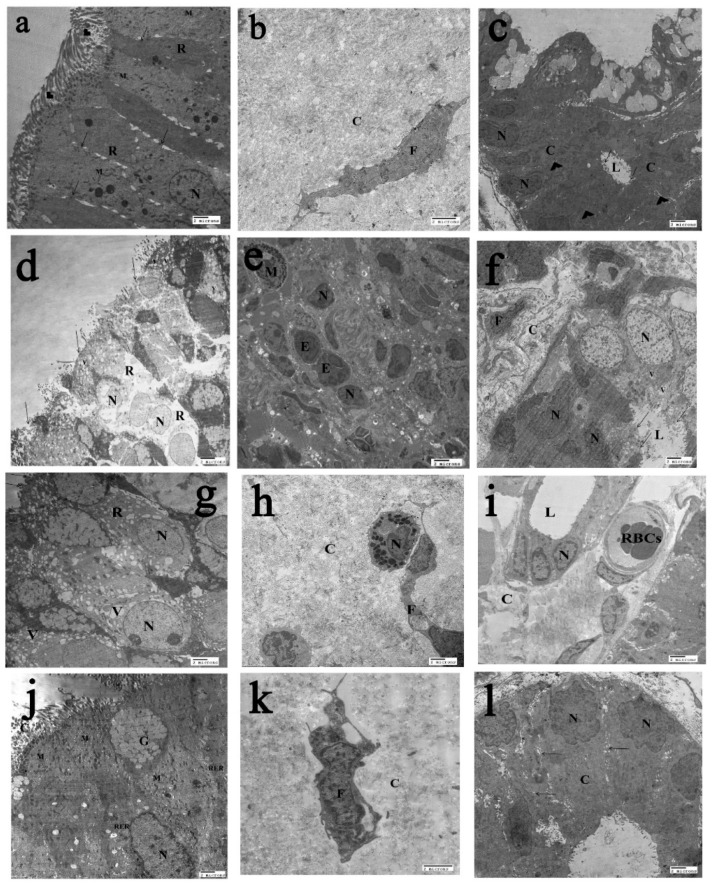
(**a**) Transmission electron micrograph of nasal epithelium surface of control group showing intact well-ciliated surface with intact desmosomes (arrow), cilia (arrow head), more or less normal mitochondria (M), rough endoplasmic reticulum (R), and regular nucleus (N). (**b**) An electron micrograph of lamina propria of nasal mucosa of control group showing organized collagen (C) and more or less normal fibroblast (F). (**c**) Transmission electron micrograph of Bowman’s gland in control group showed nasal mucosa showing normal nucleus (N), homogeneous electron dense cytoplasm (C), continuous microvilli (arrow) lining the lumen (L), and intact junctional complex between the cells (arrow head). (**d**) Transmission electron micrograph of nasal epithelium surface of AR group showing disrupted epithelial cell surface with marked loss of cilia (arrow). Severe cytoplasmic vacuolation is seen (V). Massive rarefaction of cytoplasm (R) and marked disruption of nucleus (N). (**e**) An electron micrograph of lamina propria of nasal mucosa of AR group showing infiltration with mast cell (M), eosinophil (E), and neutrophil (N). (**f**) Transmission electron micrograph of Bowman’s gland in the AR group showed nasal mucosa showing irregular nucleus (N), cytoplasmic vacuolation (V), and disrupted microvilli (arrow) lining the lumen (L). Disorganization and rarefication of collagen (C) surrounding Bowman’s gland with irregular fibroblast (F). (**g**) Transmission electron micrograph of nasal epithelium surface of the Montelukast group showing focal area of epithelial cell surface with marked loss of cilia (arrow), vacuolated cytoplasm (V), and regular round to oval nucleus (N). (**h**) An electron micrograph of lamina propria of nasal mucosa of the Montelukast group showing disorganized collagen (C) and distorted fibroblast (F). (**i**) Transmission electron micrograph of Bowman’s gland in nasal mucosa showed mostly normal nucleus (N) with intact cell junction, microvilli lining the lumen (L), focal area of rarefied collagen (C) surrounding the Bowman’s gland, and blood vessel congested with RBCs. (**j**) Transmission electron micrograph of nasal epithelium surface of the MSCs treated group showing mostly normal metabolically active cell rich in organelles, nucleus (N), cilia (C), goblet (G), mitochondria (M), and rough endoplasmic reticulum (RER). (**k**) An electron micrograph of lamina propria of nasal mucosa of the MSCs treated group showing slightly rarefied collagen (C) and more or less normal fibroblast (F). (**l**) Transmission electron micrograph of Bowman’s gland in MSCs treated nasal mucosa showing more or less normal nucleus (N), electron dense cytoplasm (C), and intact interdigitation processes (arrow). (Scale bar = 2 µm).

The ultrastructure of the control group (Group I) showed the surface epithelium of nasal mucosa with intact well-ciliated surface, more or less normal mitochondria, rough endoplasmic reticulum, and regular nucleus. The cells were closely opposed to each other by junctional complexes between the cells (tight junction and apical junctional complexes) (Figure 7a). The lamina propria showed organized collagen and more or less normal fibroblast (Figure 7b). The Bowman’s gland in nasal mucosa showed normal nucleus, homogenous electron dense cytoplasm, and continuous microvilli lining the lumen, with intact junctional complex between the cells (Figure 7c). In the AR group (Group II), the nasal surface epithelium showed disrupted epithelial cell surface with marked loss of cilia, rarefied cytoplasm with vacuolation, and marked disruption of nucleus (Figure 7d). The lamina propria of nasal mucosa showed infiltration with mast cell, eosinophil, and neutrophil (Figure 7e). The collagen fibers were disorganized and rarefied with irregular fibroblast; moreover, Bowman’s gland in nasal mucosa showed irregular nucleus and cytoplasmic vacuolation and was surrounded with irregular fibroblast (Figure 7f). Also, the AR+Montelukast group (Group III) demonstrated loss of cilia from the surface epithelium of nasal mucosa, and vacuolated cytoplasm was observed as well as regular rounded to oval nucleus (Figure 7g). The lamina propria of nasal mucosa showed disorganized collagen, distorted fibroblast, and infiltration with neutrophil (Figure 7h). Bowman’s gland in nasal mucosa showed mostly normal nucleus with intact cell junction and microvilli lining the lumen. Also, there were focal areas of rarified collagen, congested blood vessels with RBCs, and eosinophil infiltration (Figure 7I). On the other hand, the AR+MSCs group (Group IV) showed surface epithelium of nasal mucosa with intact well-ciliated surface, most probably normal metabolically active cell rich in organelles, nucleus, cilia, goblet, mitochondria, and rough endoplasmic reticulum (RER) (Figure 7j). The lamina propria of nasal mucosa showed slightly rarefied collagen and more or less normal fibroblast (Figure 7k). Bowman’s gland showed more or less normal nucleus, electron dense cytoplasm, and intact interdigitating processes (Figure 7l).

## 3. Discussion

Allergic rhinitis is a common inflammatory disease worldwide [11]. Patients with AR suffer from increased airway resistance, oral breathing difficulty, sleep disturbance, and reduced social activities [12,13]. Nowadays, chemical drugs towards AR are limited to antihistamines, antileukotrienes, and intranasal corticosteroids, which only alleviate allergic symptoms but fail to regulate the allergic reaction. Infrequently, these drugs have side-effects, such as headache, throat irritation, and nasal dryness. Thus, the development of more safe and effective therapeutic agents is needed [26]. 

MSCs are increasingly being found to have potent anti-inflammatory effects in a wide range of inflammatory and immune-mediated disease models [27]. Therefore, the immunomodulatory function of MSCs makes them promising candidates for allergic disease therapy [9]. Administration of MSCs can ameliorate the severity of acute injury and fibrosis with modulation of proinflammatory and anti-inflammatory cytokines, which is considered as the main beneficial effect of MSCs. Since AR is characterized as chronic inflammation with eosinophilic infiltration and unbalance between T_H_1- and T_H_2-derived cytokines, we propose that MSCs-driven immunomodulation contributes to attenuation of inflammation in AR, consequently improving patient lifestyle [28].

In this study, we induced allergic inflammation in a rat model using three doses of OVA subcutaneous injection (Day 1, Day 5 and Day 10) and repeated intranasal booster sensitization for seven days, which resulted in a successful allergy model with clinical symptoms of sneezing and eye/nasal rubbing in albino rats. We showed that MSCs had significant inhibitory effects on allergic inflammation, compared to the efficacy of antileukotriene, which is consistent with other studies on AR or the asthma model [28,29,30,31,32].

Since immunoglobulins play important roles in mediating inflammatory reactions and hypersensitivity, we investigated the concentration of several main immunoglobulin antibodies that have been implicated in B-cell immune responses controlled by cytokines from T_H_ cells. IgE, IgG1, IgG2a, PGE2, and histamine levels were determined. There was significant (*p* < 0.05) elevation in a T_H_2-dependent immune response indicated by an increase in the serum levels of OVA-specific IgE, IgG1, IgG2a, PGE2 and histamine in the AR group when compared with the control group (Figure 3). These findings are in accordance with several previous studies [33,34], which revealed that re-exposure to the same antigen triggers degranulation of mast cells via IgE-mediated mechanism and finally leads to an increased release of inflammatory mediators in nasal mucosa, manifested by sneezing, itching, and watery discharge [33,35]. Interestingly, treatment with MSCs had a statistically significant (*p* < 0.05) tendency to reduce the secretion of IgE, IgG1, and histamine, indicating that MSCs may downregulate T_H_2 immune responses. In contrast, IgG2a and PGE2 levels were significantly (*p* < 0.05) higher in the AR+MSC group than the AR group. This reveals the T_H_1 immune response may be priming by the mounting of IgG2a and PGE2 production [8,36]. In other words, the maintenance of IgG1 and increase of IgG2a suggest the shift of T_H_2 to T_H_1 immune response. This shift may be responsible for the reduction of allergic inflammation by MSCs injection.

Given the reciprocal regulatory characteristic of T_H_1- and T_H_2-associated cytokines, we also tested the levels of mRNA expression of several interleukins and interferon in nasal tissue (Figure 4). The AR group showed significant (*p* < 0.05) upregulation in IL-4, TNF-α, TGF-β, VCAM-1 and CCL11, when compared with control rats. These findings have been confirmed by previous models of AR. Samivel et al., 2015 [15] found that mRNA expression of nasal cytokine in the OVA-challenged mice increased IL-4, IL-5, and IL-6 and decreased IFN-γ mRNA expression levels in the nasal mucosa. Other researchers showed significantly increased levels of IL-4, IL-5, and IL-13 with a significant decrease in TGF-β in the OVA-challenged mice [28]. Although TNF-α is associated predominantly with T_H_1-mediated inflammation, it is required for the production of the T_H_2-type cytokines, and is necessary for the homing of T_H_2 cells to the site of allergic inflammation. TNF-α is released in allergic responses to both mast cells and macrophages through IgE-dependent mechanisms. IgE production is induced mainly by IL-4. Several previous studies demonstrated that TNF-α enhanced the effect of IL-4 on IgE production. Indeed, TNF-α has an important role in the expression of adhesion molecules that induce transendothelial migration of eosinophils [37,38]. 

TGF-β has multiple regulatory effects on T cell proliferation, antigen presentation, and expression of costimulatory proteins, and it is relevant to the function of T regulatory cells. TGF-β levels of AR patients were detected as lower than those of controls [39]. This is, however, different from our results, as the upregulation of mRNA expression of TGF-β in our experiment is mild. On the other hand, it is plausible to find upregulation of CCL11 in the AR group, since CCL11 has been pointed out as one of the major chemokines involved in recruiting circulating eosinophils to allergic airway tissues [40,41]. Furthermore, eosinophil migration, which is dependent on the expression of cytokines, chemokines, and adhesion molecules (vascular cell adhesion molecule-1 (VCAM-1)), plays a key role in allergic rhinitis, as well as in IgE-mediated allergy [42].

The clinical and biochemical findings of the AR group in the present study were confirmed histologically by light microscope examination (Figure 5), which revealed damage of respiratory epithelium resting on apparently thick basal lamina and lamina propria and infiltration of numerous inflammatory cells with marked collagen fibers deposition confirmed by Masson’s Trichrome staining (Figure 6). 

The OVA allergen administration caused significant changes in the structure of the nasal mucosa, as the epithelial cells lost their positions, mucosal exfoliation occurred, and eosinophils infiltrated the basal stromal layer [33]. Different AR rat models showed damaged nasal epithelium with intense inflammatory cells infiltration (plasma cells, lymphocytes, and eosinophils) in the epithelium and sub epithelium [43]. Moreover, Knipping et al., 2009 [44] revealed that the nasal mucosa of AR patients had disintegrated epithelium with many goblet cells, edematous lamina propria with dilated capillaries and sever inflammatory infiltration. Knipping and his colleagues connected these histological findings with cytokines pattern in AR, as after sensitization with an allergen, IgE mediated cytokines released like leukotrienes, and histamine caused by massive infiltration of nasal mucosa with mast cells, eosinophilic granulocytes, and active plasma cells, which have extensive endoplasmic reticulum. 

The electron microscope findings of the current study confirm the light microscopical findings, which revealed disrupted epithelial cell surface with marked loss of cilia, rarefied cytoplasm, and infiltration of lamina propria with mast cell, eosinophil and neutrophil. Also, the collagen fibers were disorganized and rarefied with irregular fibroblast, with Bowman’s gland in nasal mucosa showing irregular nucleus and cytoplasmic vacuolation, surrounding Bowman’s gland with irregular fibroblast (Figure 7).

The ultrastructure of nasal mucosa in patients with AR revealed that the nasal mucosal epithelium had increased ciliary loss with swelling of reaming cilia, few microvilli, widening of intercellular spaces, and eosinophilic infiltration. Also, the sub-epithelial zone had irregular arrangement of collagen fibers with many types of inflammatory cells [45]. Moreover, Knipping and his colleagues [44] showed that the ultrastructure of nasal mucosa from patients with AR revealed that the capillaries were dilated with active and elevated endothelium with many pinocytotic vesicles. Also, the sub-epithelial mucosa had activated plasma cells with marked endoplasmic reticulum and mast cells with typically thickened granules and partly emptied vesicles. 

Gene expressions of IL-4, TNF-α, VCAM-1 and CCL11 were significantly decreased and TGF-*β* significantly increased in the AR + Montelukast group compared to the AR group. In accordance with these results, Saito et al., 2004 [46], showed that the numbers of eosinophils in the nasal mucosa were significantly decreased in patients with AR treated with Montelukast. High doses of montelukast reduced serum IgE levels and bronchoalveolarlavage (BAL) IL-4 and IL-5 levels in OVA-sensitized BALB/c mice challenged with the inhalation of OVA [47]. Moreover, the attachment, adhesion, and transendothelial migration of eosinophils to the site of inflammation are upregulated by Th2 cytokines by inducing the expression of intercellular adhesion molecule-1 (ICAM-1), VCAM-1, and E-selectin on the vascular endothelium. TNF-α, generated from inflammatory cells in response to IL-4, may induce the expression of VCAM-1 that subsequently activates certain subsets of leukocytes, resulting in both increased expression and prolonged appearance of VCAM-1 on the endothelium [48]. These findings were confirmed histologically by light microscope, which revealed partial damage of respiratory epithelium rests on thick basal lamina and lamina propria with infiltration of a few inflammatory cells. Masson’s trichrome staining revealed collagen fibers accumulation at basal lamina and lamina propria was minimally decreased. Also, the electron microscopic examination of AR + Montelukast group demonstrated marked loss of cilia, vacuolated cytoplasm, disorganized collagen, and distorted fibroblast. 

In accordance with these results, Sun and his colleagues [29] revealed that administration of Montelukast alone in a guinea pig model for allergic rhinitis (AR) failed to prevent the allergic rhinitis symptoms while with the combination therapy of Montelukast and sodium chromoglycate, the AR symptoms (sneezing and rubbing frequencies) were significantly prevented with marked prevention of inflammatory cells infiltration into the nasal mucosa. Also, the histological structure of nasal mucosa treated only with Montelukast showed partial damage of respiratory epithelium with decreased inflammatory cell infiltration and thick lamina propria. Moreover, the cysteinyl leukotrienes (CysLTs) promote inflammatory cells recruitment and activation (primarily of eosinophils) as well as fibrosis and airway remodeling, with actions such as smooth muscle and epithelial cells proliferation. The CysLTs increase expression of adhesion molecules such as VCAM-1 associated with eosinophilia as a result of reducing eosinophil apoptosis. The CysLTs may also promote airway remodeling by increasing the deposition of collagen below the basement membrane, enhancing collagen synthesis and degradation by fibroblasts, and promoting the proliferation of bronchial epithelial cells and smooth muscle cells [49].

Interestingly, we found that treatment of AR with MSCs (AR + MSCs group) induced significant down-regulations in the gene expressions of IL-4, CCL-11, VCAM, and TNF-α, with a significant increase in the levels of TGF-β when compared with AR group. Chunlei et al., 2017 [33], showed that the levels of histamine, IgE, and IL-4 were dramatically decreased by the MSCs treatment in AR rat model. Furthermore, the level of TNF-α was decreased significantly after the MSCs treatment. Therefore, they claimed that MSCs play a role in the regulation of the production of inflammatory cytokines, in particular, IL-4 and TNF-α. Also, Cho with his colleges [28] found that the systemic administration of MSCs enhances Tregs expansion and gene expression of TGF-β and PGE2. 

In a rat model of AR treated with multiple dosages of MSCs, the nasal mucosal tissues exhibited nearly no histological alterations compared with normal nasal mucosal tissues confirmed by standard histology scoring system suggesting that multiple administration of MSCs inhibits epithelial tissues damage caused by AR [33]. Moreover, Zhao et al., 2016 [50], assessed the potential of bone marrow-derived mesenchymal stem cells (BMSCs) on AR mouse model revealing that multiple dosage administration of BMSCs improve the histological changes of AR including decrease of epithelium thickness, goblet, mast cells, and eosinophil cells infiltration. 

These findings were confirmed histologically by light microscope, which revealed that nasal mucosa was near normal with overcrowded respiratory epithelium resting on thin basal lamina and lamina propria of loose connective tissue with infiltration of a few cells. Also, there was a significant decrease in collagen fibers deposition confirmed by Masson’s trichrome staining. The electron microscope examination of Group IV revealed that the surface epithelium of nasal mucosa was intact and well-ciliated with mostly normal metabolically active cell and with less collagen fibers and normal fibroblast. In agreement with our findings, Samivel et al., 2015 [15] found that the nasal rubbing and sneezing scores were significantly decreased in the MSCs treated groups in AR mice model. The immuno-expression of CCL11 and VCAM-1 was significantly decreased in groups treated with MSCs compared to AR mice model. Also, the total IgE, OVA-specific IgE, and IgG1 levels were significantly decreased; however, the OVA-specific IgG2a level did not change significantly in the MSCs-treated groups compared with the positive control group. 

It has been reported that MSCs mediate their immunomodulatory effects by interacting with adaptive immunity systems and remolding the altered T_H_1/ T_H_2 balance in allergic rhinitis. The main mechanisms of MSCs in immunomodulation are T-cell suppression by cell-cell contact, release of soluble factors, and generation of regulatory lymphocytes. Concerning cell-to-cell contact, the MSCs express integrins (alpha1, alpha2, alpha3, alpha5, alpha6, alpha v, beta 1, beta3, and beta4), intercellular adhesion molecules (ICAM-1, ICAM-2), vascular cell adhesion molecule (VCAM-1), CD72, and CD58 (LFA-3), on their surface, thus being able to bind to T lymphocytes with high affinity. Also, the T-cell attraction process by MSC can be explained by the expression of high levels of several leukocyte chemokines such as chemokine (C-X-C motif) ligand 9 (CXCL9), CXCL10, and CXCL11 [51]. MSCs may exert their immunomodulation by releasing soluble factors [52]; however, the production of suppressive soluble factors is dependent on a cross talk between MSCs and activated T-cells. There are more than 30 soluble factors associated with the immunomodulation capacity on T-lymphocyte activation and proliferation by MSCs [53], such as hepatic growth factor (HGF), TGF-β, indoleamine 2,3-dioxygenase (IDO), PGE2, nitric oxide (NO), IL-6, IL-10 [54].

## 4. Materials and Methods

### 4.1. Experimental Animals

Adult male albino rats (180–200 g), 6 weeks of age, were purchased from the Experimental Animal Unit, Faculty of Veterinary Medicine, Benha University, Egypt. The rats were bred and maintained in an air-conditioned animal house under specific pathogen-free conditions. All animals were housed in clean cages and given a standard diet and clean water ad libitum. The rats were subjected to a normal light/dark cycle (12-h light-dark cycle starting at 8:00 AM) and room temperature (23 ± 3 °C) and allowed free access to chow and water. This study was carried out in strict accordance with the recommendations in the Guide for the Care and Use of Laboratory Animals of the National Institutes of Health (NIH publication No. 85–23, revised 2011). All protocols were approved by the institutional review board for animal experiments of the Faculty of Medicine, Benha University, Egypt (BUFM 3 January 2018).

### 4.2. Ovalbumin-induced Allergic Rhinitis Rat Model 

The rats were sensitized by OVA by using a standard protocol. Briefly, ovalbumin (OVA, grade V, Sigma, St. Louis, MO, USA) and aluminum hydroxide gel (Sigma), diluted in normal saline (1 mg/mL + 30 mg/mL + 1 mL), were used to sensitize the rats 3 times via subcutaneous injection in 5 sites (double back toes, bilateral inguinal subcutaneous region, and abdominal cavity, with 0.2 mL for each site) on days 1, 5, and 10. From days 15 to 21, the sensitized rats were intranasally challenged with daily drops of OVA diluted with sterile physiological saline (50 μL/nostril, 100 mg/mL, 10% OVA).

### 4.3. Experimental Design and Treatment Protocol

**Figure 8:**
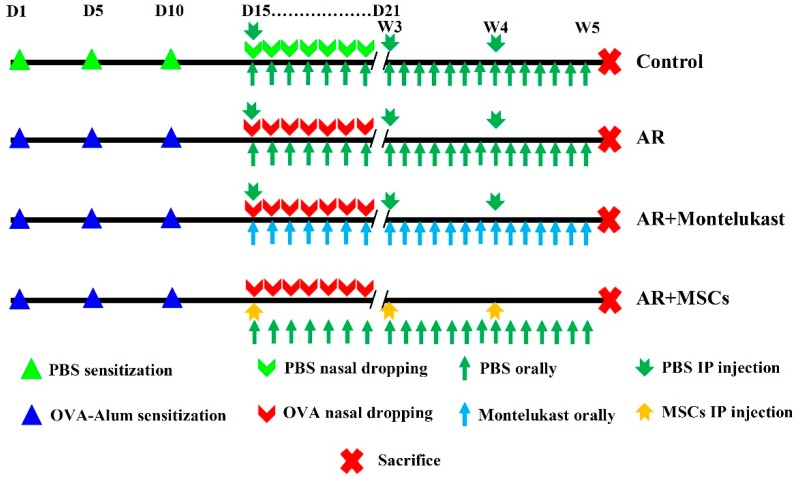
Experimental design and treatment procedure. The rats were divided into four groups: Group I (control group; *n* = 15 subdivided into 3 subgroups [5 rats each]), Group II (AR group; *n* = 6), Group III (AR + Montelukast; *n* = 6), and Group IV (AR + MSCs; *n* = 6). The rats of the AR groups were sensitized by OVA and then challenged with daily nasal drops of OVA diluted in sterile physiological saline (50 μL/nostril, 100 mg/mL, 10% OVA) from days 15 to 21 with/without treatment by Montelukast (1 h before each challenge) or MSCs I/P injection (1 × 10^6^ MCSs; weekly for 3 constitutive weeks). Both Montelukast and MSCs treatment started from day 15 of the experiment. At the end of the 5th week, blood samples were collected from all rats for immunological assays and histological and molecular biology examinations.

Thirty-three rats were divided randomly into four groups (Figure 8) as follows:

**Group I (control group; *n* = 15):** The rats were fed a regular chow diet. The rats were divided into 3 subgroups; each subgroup contained 5 rats as follows:

**Subgroup a:** 5 rats were left without any intervention.

**Subgroup b**: 5 rats were injected with normal saline into 5 sites (double back toes, bilateral inguinal subcutaneous region, and abdominal cavity, with 0.2 mL for each site) and the peritoneum (on days 1, 5, and 10). Then, the rats were intranasally challenged with daily nasal drops of normal saline from days 15 to 21.

**Subgroup c**: 5 rats were intraperitoneally injected with phosphate buffered saline solution weekly for 3 constitutive weeks from day 15 of the experiment.

**Group II (AR group; *n* = 6):** The rats were sensitized by OVA and then challenged with daily nasal drops of OVA diluted with sterile physiological saline (50 μL/nostril, 100 mg/mL, 10% OVA) from day 15 to day 20 of the experiment and then daily till the end of the experiment.

**Group III (AR + Montelukast; *n* = 6)**: The rats in the OVA-induced AR model were given Montelukast sodium at 5.0 mg/kg diluted in 150 µL of sterile water by gavage, using an oral administration tube (animal feeding tube, Popper^®^; Fisher Scientific, Nepean, ON, Canada) 1 h before each challenge from the 15th day of the experiment for 6 days (15th–20th), and then daily till the end of the experiment. 

**Group IV (AR + MSCs group; *n* = 6):** The rats in the OVA-induced AR model underwent intraperitoneal MSCs injection (1 × 10^6^ MCSs were suspended in 0.5 mL of phosphate buffered saline) weekly for 3 constitutive weeks, starting from day 15 day of the experiment.

### 4.4. Sampling

At the end of the 5th week, the rats were anesthetized by sodium thiopental (40 mg/kg I.P.) after 12 h of fasting. The rats were fixed on an operating table and blood samples were obtained from retro-orbital venous plexus using a fine-walled Pasteur pipette. Serum and plasma were obtained following rapid centrifugation and stored at −80 °C. Then vascular perfusion fixation through the left ventricle was performed. The heads of rats from each group were removed, after removing the nasal cavity from the head of the remaining rat; the nasal mucosa was meticulously removed by using a small curette. The nasal mucosa was collected from rats of all groups for light microscope and transmission electron microscopy examinations and for qPCR study.

### 4.5. MSCs Isolation, Culture, and Tracking

#### 4.5.1. Adipose Derived Stem Cell Preparation

The adipose tissues from abdominal wall of rats were obtained and then placed into a labeled sterile tube containing 15 mL of a phosphate buffered solution (PBS; Gibco/Invitrogen, Grand Island, NY, USA). Enzymatic digestion was performed using 0.075% collagenase II (SERVA Electrophoresis GmbH, Heidelberg, Germany) in Hank’s Balanced Salt Solution for 60 min at 37 °C with shaking. Digested tissue was filtered and centrifuged, and erythrocytes were removed by treatment with an erythrocyte lysis buffer. The cells were transferred to tissue culture flasks with Dulbecco Modified Eagle Medium (DMEM, Gibco/ BRL, Grand Island, New York, USA) supplemented with 10% fetal bovine serum (FBS) (Gibco/BRL) and, after an attachment period of 24 h, non-adherent cells were removed by a PBS wash. Attached cells were cultured in DMEM supplemented with 10% FBS, 1% penicillin-streptomycin (Gibco/BRL), and 1.25 mg/L amphotericin B (Gibco/BRL), and expanded in vitro. At 80–90% confluence, cultures were washed twice with PBS and the cells were trypsinized with 0.25% trypsin in 1 mM EDTA (Gibco/BRL) for 5 min at 37 °C. After centrifugation, cells were resuspended in serum-supplemented medium and incubated in 50 cm^2^ culture flask (Falcon). The resulting cultures were referred to as first-passage cultures and expanded in vitro until passage three [55]. Furthermore, the adipocytes differentiation was achieved by adipocytes StemPro® adipogenesis differentiation kit (Gibco, Life Technology, Carlsbad, CA, USA) and they were stained by Oil Red O stain (Sigma-Aldrich, St Louis, MO, USA). The osteocytes differentiation was achieved by the osteocytes StemPro® osteogenesis differentiation kit (Gibco, Carlsbad, CA, USA) and stained by Alizarin Red S stain (Sigma-Aldrich Chemical Co., St. Louis, MO, USA).

#### 4.5.2. Labeling stem cells with Green Fluorescent Protein (GFP)

MSCs labeled with GFP were observed in tissue culture using a fluorescence microscope (Leica Microsystems CMS GmbH, Ernst-Leitz-Straße, Wetzlar, D-35578, Germany) [56]. MSCs were transfected with non-integrating plasmids containing GFP. One day prior to transfection, 5 × 10^5^ cultured cells were plated in 1 mL complete growth medium and allowed to grow until the cells were 50–70% confluent at the time of transfection. MSCs were transfected with a single plasmid using the Nucleofector kit (Lonza, Verviers, Belgium) according to the manufacturer’s instructions.

#### 4.5.3. Immunophenotyping Characterization of Differentiated Stem Cells

MSCs in culture were characterized by their adhesiveness and fusiform shape and by detection of CD45 and CD29 (surface markers of rat mesenchymal stem cell) by flow cytometry. Cells were trypsinized and adjusted to 1 × 10^6^ cells/mL. Then 1 × 10^5^ cells were incubated with 10 μL of monoclonal antibodies: CD45 PE and CD29 PE, at 4 °C in the dark; the same species isotypes served as a negative control. After 20 min incubation, 2 mL of PBS containing 2% FCS solution was added to each tube of monoclonal-treated cells. The mixtures were then centrifuged for 5 min at 2500 rpm followed by discarding of the supernatant and resuspending of the cells in 500 μL PBS containing 2% FCS. Cell analysis was performed using CYTOMICS FC 500 Flow Cytometer (Beckman Coulter, Brea, CA, USA) and CXP software version 2.2 [57].

#### 4.5.4. Evaluation of Nasal Symptoms

A day before the end of the experiment (4 weeks after the first MSCs injection), the rats were stimulated by nasal drops of OVA (20 μL/nostril, 50 mg/mL) dissolved in a physiological saline solution into the bilateral nasal cavities and maintained in the observation cage. A blinded observer recorded the instances of sneezing and nose scratching for 10 min after the OVA intranasal stimulation to evaluate the degree of allergic responses. The control rats were processed with saline.

#### 4.5.5. Estimation of Serum OVA-Specific IgE, IgG1, IgG2a, PGE2 and Histamine

The serum levels of OVA-specific immunoglobulin E (IgE), IgG1, IgG2a, PGE2 and histamine were measured by enzyme-linked immunosorbent assay (ELISA) (USCN Life Science, Houston, TX, USA). 

### 4.6. Determination of the Expressions of Interleukin (IL)-4, Transforming Growth Factor (TGF)-β, Tumor Necrosis Factor (TNF)-α, Vascular Cell Adhesion Molecule-1 (VCAM-1) and C-C Motif Chemokine Ligand 11 (CCL11) Genes by qPCR

#### 4.6.1. Total RNA Extraction and Reverse Transcription

Total RNA was extracted from frozen nasal tissue samples by the Trizol method (Invitrogen, Carlsbad, CA, USA) using RNeasy mini kit (Qiagen, Hilden, Germany) as previously described [58]. Samples were quantified using NanoDrop One spectrophotometer (Thermo Fisher Scientific, Inc., Waltham, MA, USA). RNA (1 µg) was reverse transcribed by T100 Thermal Cycler (BioRad, Hercules, CA, USA) using Maxima First Strand cDNA Synthesis Kit (Thermo Fisher Scientific, USA), following the manufacturer’s guidelines [59].

#### 4.6.2. Quantitative real-time PCR

Real-time PCR was performed according to the manufacturer’s instructions using Maxima SYBR Green/ROX qPCR Master Mix (Thermo Fisher, USA), by Step One Plus Real-Time PCR System (Life Technologies, USA) [60]. The primers sequences were as follows: **GAPDH forward**: TGATTCTACCCACGGCAAGTT; **GAPDH reverse**: TGATGGGTTTCCCATTGATGA; IL-4 **forward**: CCAACTGCTTCCCCCTCTG; **IL-4 reverse**: TCTGTTACGGTCAACTCGGTG; **TGF-β forward**: GCGGCAGCTGTACATCGA; **TGF-β reverse**: GGCTCGTGAATCCACTTCCA; TNF-α **forward:** AACTCCAGCCGGTGCCTAT; TNF-α **reverse:** GTTCAGCAGGCAGAAGAGGATT; **VCAM-1 forward:** TGACAAGTCCCCATCGTTGA; **VCAM-1 reverse:** ACCTCGCGACGGCATAATT; **CCL11 forward:** ATACCCCTTCAGCGACTAGAG and **CCL11 reverse**: GCTTTGGAGTTGGAGATTTTTGG. The mRNA expression of each sample was determined after correction by GAPDH expression. The relative expression was calculated using the 2^−ΔΔ*C*T^ method. The results were expressed as the n-fold difference relative to the control group. Each sample was assayed three times.

### 4.7. Histopathological Analysis

#### 4.7.1. Light Microscopic Study

The specimens were excised; paraffin sections of 4–6 µm thickness were performed and then mounted on glass slides for H & E and Masson’s trichrome stains [61].

#### 4.7.2. Morphometric Study

The mean area percent of collagen fibers deposition by Masson’s trichrome was quantified in five images from five non-overlapping fields from each rat of each group using Image-Pro Plus program version 6.0 (Media Cybernetics Inc., Bethesda, Maryland, USA).

#### 4.7.3. Transmission Electron Microscopic Study 

Vascular perfusion fixation through the left ventricle with 1% glutaraldehyde was performed; then, the nasal mucosa was dissected and 1 mm^3^ of nasal mucosa samples was taken. The samples were fixed with 2.5% buffered glutaraldehyde in 0.1 M phosphate buffer solution (PBS) of pH 7.4 at 4 °C for 2 h and then washed three times with PBS (10 min each). The samples were then post-fixed in 1% osmic acid for 30 min and then washed three times with PBS (10 min each). Samples were dehydrated with ascending series of ethyl alcohol (30, 50, 70, 90% and absolute alcohol), each concentration for 30 min. The samples were infiltrated with acetone for 1 h and then embedded in Araldite 502 resin. The plastic molds were cut using LEICA Ultra cut (UCT ultra-microtome) and stained with 1% toluidine blue. After examination of semi-thin sections, ultra-thin sections (50–60 nm thick) were cut, stained with uranyl acetate, then counter-stained with lead citrate, examined and photographed using JEOL-JEM-100 SX electron microscope, Electron Microscope Unit, Tanta University [62].

### 4.8. Statistical Analysis

A statistical analysis was performed using the statistical software package SPSS for Windows (version 18.0; SPSS Inc., Chicago, IL, USA). Differences between groups were evaluated using a one-way ANOVA followed by *Duncan* post-hoc test. For each test, all the data are expressed as the mean ± standard error of mean (SE), and a *p* value <0.05 was considered significant.

## 5. Conclusions

Administration of adipose tissue-derived MSCs effectively reduced allergic symptoms and inflammatory parameters in the rat model of AR through reduction of T_H_2 cytokines and OVA-specific IgE secretion from B cells. Therefore, MSC treatment is potentially an alternative therapeutic modality in AR.

## Abbreviations

AR	Allergic rhinitis
CCL11	C-C Motif Chemokine Ligand 11
ICAM	Intercellular adhesion molecules
Ig	Immunoglobulin
IL-	Interleukin-
MSCs	Mesenchymal stem cells
OVA	Ovalbumin
PGE2	Prostaglandin E2
TGF-	Transforming growth factor-
TNF-	Tumor necrosis factor-
VCAM	Vascular cell adhesion molecule

## References

[1] Singer N.G., Caplan A.I. (2011). Mesenchymal stem cells: Mechanisms of inflammation. Annu. Rev. Pathol. Mech. Dis..

[2] Turker Sener L., Albeniz I. (2015). Challenge of mesenchymal stem cells against diabetic foot ulcer. Curr. Stem Cell Res. Ther..

[3] Yang F.Y., Chen R., Zhang X., Huang B., Tsang L.L., Li X., Jiang X. (2018). Preconditioning Enhances the Therapeutic Effects of Mesenchymal Stem Cells on Colitis Through PGE2-Mediated T-Cell Modulation. Cell Transplant..

[4] Gaber T., Schönbeck K., Hoff H., Tran C., Strehl C., Lang A., Ohrndorf S., Pfeiffenberger M., Röhner E., Matziolis G. (2018). CTLA-4 Mediates Inhibitory Function of Mesenchymal Stem/Stromal Cells. Int. J. Mol. Sci..

[5] Uccelli A., Moretta L., Pistoia V. (2008). Mesenchymal stem cells in health and disease. Nat. Rev. Immunol..

[6] Uccelli A., Pistoia V., Moretta L. (2007). Mesenchymal stem cells: A new strategy for immunosuppression?. Trends Immunol..

[7] Bonfield T.L., Koloze M., Lennon D.P., Zuchowski B., Yang S.E., Caplan A.I. (2010). Human mesenchymal stem cells suppress chronic airway inflammation in the murine ovalbumin asthma model. Am. J. Physiol. Lung Cell. Mol. Physiol..

[8] Fu Q., Chow Y., Sun S., Zeng Q., Li H., Shi J., Sun Y., Wen W., Tse H., Lian Q. (2012). Mesenchymal stem cells derived from human induced pluripotent stem cells modulate T-cell phenotypes in allergic rhinitis. Allergy.

[9] Fan X.-L., Zeng Q.-X., Li X., Li C.-L., Xu Z.-B., Deng X.-Q., Shi J., Chen D., Zheng S.G., Fu Q.-L. (2018). Induced pluripotent stem cell-derived mesenchymal stem cells activate quiescent T cells and elevate regulatory T cell response via NF-κB in allergic rhinitis patients. Stem Cell Res. Ther..

[10] Yang C., Li J., Lin H., Zhao K., Zheng C. (2015). Nasal mucosa derived-mesenchymal stem cells from mice reduce inflammation via modulating immune responses. PLoS ONE.

[11] Han D., Wang C., Lou W., Gu Y., Wang Y., Zhang L. (2010). Allergen-specific IL-10-secreting type IT regulatory cells, but not CD4+ CD25+ Foxp3+ T cells, are decreased in peripheral blood of patients with persistent allergic rhinitis. Clin. Immunol..

[12] Stuck B.A., Czajkowski J., Hagner A.-E., Klimek L., Verse T., Hörmann K., Maurer J.T. (2004). Changes in daytime sleepiness, quality of life, and objective sleep patterns in seasonal allergic rhinitis: A controlled clinical trial. J. Allergy Clin. Immunol..

[13] Maurer M., Zuberbier T. (2007). Undertreatment of rhinitis symptoms in Europe: Findings from a cross-sectional questionnaire survey. Allergy.

[14] Licari A., Ciprandi G., Marseglia A., Castagnoli R., Barberi S., Caimmi S., Marseglia G.L. (2014). Current recommendations and emerging options for the treatment of allergic rhinitis. Expert Rev. Clin. Immunol..

[15] Samivel R., Kim E.H., Chung Y.-J., Mo J.-H. (2015). Immunomodulatory effect of tonsil-derived mesenchymal stem cells in a mouse model of allergic rhinitis. Am. J. Rhinol. Allergy.

[16] Galli S.J., Tsai M., Piliponsky A.M. (2008). The development of allergic inflammation. Nature.

[17] Gould H.J., Sutton B.J. (2008). IgE in allergy and asthma today. Nat. Rev. Immunol..

[18] Takhar P., Corrigan C.J., Smurthwaite L., O’connor B.J., Durham S.R., Lee T.H., Gould H.J. (2007). Class switch recombination to IgE in the bronchial mucosa of atopic and nonatopic patients with asthma. J. Allergy Clin. Immunol..

[19] Eifan A., Durham S. (2016). Pathogenesis of rhinitis. Clin. Exp. Allergy.

[20] Min Y.-G. (2010). The pathophysiology, diagnosis and treatment of allergic rhinitis. Allergy Asthma Immunol. Res..

[21] Mandhane S.N., Shah J.H., Thennati R. (2011). Allergic rhinitis: An update on disease, present treatments and future prospects. Int. Immunopharmacol..

[22] Peters-Golden M., Henderson W.R. (2005). The role of leukotrienes in allergic rhinitis. Ann. Allergy Asthma Immunol..

[23] Elnabtity M.H., Singh R.F., Ansong M.A., Craig T.J. (1999). Leukotriene modifiers in the management of asthma. J. Am. Osteopath. Assoc..

[24] Shirasaki H., Himi T. (2016). Role of cysteinyl leukotrienes in allergic rhinitis. Excellence in Otolaryngology.

[25] Scadding G.K. (2009). Allergic rhinitis: Background, symptoms, diagnosis and treatment options. Nurs. Times.

[26] Ricketti P.A., Alandijani S., Lin C.H., Casale T.B. (2017). Investigational new drugs for allergic rhinitis. Expert Opin. Investig. Drugs.

[27] Rasmusson I. (2006). Immune modulation by mesenchymal stem cells. Exp. Cell Res..

[28] Cho K.-S., Park M.-K., Kang S., Park H.-Y., Hong S.-L., Park H.-K., Yu H.-S., Roh H.-J. (2014). Adipose-derived stem cells ameliorate allergic airway inflammation by inducing regulatory T cells in a mouse model of asthma. Mediat. Inflamm..

[29] Sun Y.Q., Deng M.X., He J., Zeng Q.X., Wen W., Wong D.S., Tse H.F., Xu G., Lian Q., Shi J. (2012). Human pluripotent stem cell-derived mesenchymal stem cells prevent allergic airway inflammation in mice. Stem Cells.

[30] Desai M.B., Gavrilova T., Liu J., Patel S.A., Kartan S., Greco S.J., Capitle E., Rameshwar P. (2013). Pollen-induced antigen presentation by mesenchymal stem cells and T cells from allergic rhinitis. Clin. Transl. Immunol..

[31] Goodwin M., Sueblinvong V., Eisenhauer P., Ziats N.P., LeClair L., Poynter M.E., Steele C., Rincon M., Weiss D.J. (2011). Bone marrow-derived mesenchymal stromal cells inhibit Th2-mediated allergic airways inflammation in mice. Stem Cells.

[32] Cho K.S., Park H.K., Park H.Y., Jung J.S., Jeon S.G., Kim Y.K., Roh H.J. (2009). IFATS collection: Immunomodulatory effects of adipose tissue-derived stem cells in an allergic rhinitis mouse model. Stem Cells.

[33] Min Y.G. (2013). Pathophysiology, diagnosis, and treatment of allergic rhinitis. Korean J. Otorhinolaryngol. Head Neck Surg..

[34] Li C., Fu Y., Wang Y., Kong Y., Li M., Ma D., Zhai W., Wang H., Lin Y., Liu S. (2017). Mesenchymal stromal cells ameliorate acute allergic rhinitis in rats. Cell Biochem. Funct..

[35] Santos C.B., Pratt E.L., Hanks C., McCann J., Craig T.J. (2006). Allergic rhinitis and its effect on sleep, fatigue, and daytime somnolence. Ann. Allergy Asthma Immunol..

[36] Huber V.C., McKeon R.M., Brackin M.N., Miller L.A., Keating R., Brown S.A., Makarova N., Perez D.R., MacDonald G.H., McCullers J.A. (2006). Distinct contributions of vaccine-induced immunoglobulin G1 (IgG1) and IgG2a antibodies to protective immunity against influenza. Clin. Vaccine Immunol..

[37] Iwasaki M., Saito K., Takemura M., Sekikawa K., Fujii H., Yamada Y., Wada H., Mizuta K., Seishima M., Ito Y. (2003). TNF-α contributes to the development of allergic rhinitis in mice. J. Allergy Clin. Immunol..

[38] Aggarwal S., Pittenger M.F. (2005). Human mesenchymal stem cells modulate allogeneic immune cell responses. Blood.

[39] Qiu Q., Lu H., Lu C., Chen S., Han H. (2012). Variations in TGF-beta, IL-10, and IL-17 after specific immunotherapy and correlations with symptoms in patients with allergic rhinitis. J. Investig. Allergol. Clin. Immunol..

[40] Lloyd C. (2002). Chemokines in allergic lung inflammation. Immunology.

[41] Salib R., Lau L., Howarth P. (2005). Nasal lavage fluid concentrations of eotaxin-1 (CCL11) in naturally occurring allergic rhinitis: Relationship to disease activity, nasal luminal eosinophil influx, and plasma protein exudation. Clin. Exp. Allergy.

[42] Lee B.-J., Naclerio R.M., Bochner B.S., Taylor R.M., Lim M.C., Baroody F.M. (1994). Nasal challenge with allergen upregulates the local expression of vascular endothelial adhesion molecules. J. Allergy Clin. Immunol..

[43] Bozkurt M.K., Tülekb B., Bozkurt B., Akyürek N., Mehmet Ö., Kiyici A. (2014). Comparison of the efficacy of prednisolone, montelukast, and omalizumab in an experimental allergic rhinitis model. Turk. J. Med. Sci..

[44] Knipping S., Holzhausen H., Riederer A., Schrom T. (2009). Allergic and idiopathic rhinitis: An ultrastructural study. Eur. Arch. Oto-Rhino-Laryngol..

[45] Toppozada H.H., Talaat M.A. (1976). The allergic nasal mucosa following vidian neurectomy. ORL.

[46] Saito H., Morikawa H., Howie K., Crawford L., Baatjes A.J., Denburg E., Cyr M.M., Denburg J.A. (2004). Effects of a cysteinyl leukotriene receptor antagonist on eosinophil recruitment in experimental allergic rhinitis. Immunology.

[47] Wu A., Chik S., Chan A., Li Z., Tsang K., Li W. (2003). Anti-inflammatory effects of high-dose montelukast in an animal model of acute asthma. Clin. Exp. Allergy.

[48] Muller W. (2013). Getting leukocytes to the site of inflammation. Vet. Pathol..

[49] Çobanoğlu B., Toskala E., Ural A., Cingi C. (2013). Role of leukotriene antagonists and antihistamines in the treatment of allergic rhinitis. Curr. Allergy Asthma Rep..

[50] Zhao N., Liu Y., Liang H., Jiang X. (2016). Bone marrow-derived mesenchymal stem cells reduce immune reaction in a mouse model of allergic rhinitis. Am. J. Transl. Res..

[51] Haddad R., Saldanha-Araujo F. (2014). Mechanisms of T-cell immunosuppression by mesenchymal stromal cells: What do we know so far?. BioMed Res. Int..

[52] Zhao Q., Ren H., Han Z. (2016). Mesenchymal stem cells: Immunomodulatory capability and clinical potential in immune diseases. J. Cell. Immunother..

[53] Thakare V.N., Osama M., Naik S.R. (2013). Therapeutic potential of curcumin in experimentally induced allergic rhinitis in guinea pigs. Int. Immunopharmacol..

[54] Arsenijevic A., Harrell C.R., Fellabaum C., Volarevic V. (2017). Mesenchymal Stem Cells as New Therapeutic Agents for the Treatment of Primary Biliary Cholangitis. Anal. Cell. Pathol..

[55] Weiss M.L., Medicetty S., Bledsoe A.R., Rachakatla R.S., Choi M., Merchav S., Luo Y., Rao M.S., Velagaleti G., Troyer D. (2006). Human umbilical cord matrix stem cells: Preliminary characterization and effect of transplantation in a rodent model of Parkinson’s disease. Stem Cells.

[56] Niki H., Hosokawa S., Nagaike K., Tagawa T. (2004). A new immunofluorostaining method using red fluorescence of PerCP on formalin-fixed paraffin-embedded tissues. J. Immunol. Methods.

[57] Sabry D., Noh O., Samir M. (2016). Comparative evaluation for potential differentiation of endothelial progenitor cells and mesenchymal stem cells into endothelial-like cells. Int. J. Stem Cells.

[58] Chomczynski P., Sacchi N. (1987). Single-step method of RNA isolation by acid guanidinium thiocyanate-phenol-chloroform extraction. Anal. Biochem..

[59] Fleige S., Pfaffl M.W. (2006). RNA integrity and the effect on the real-time qRT-PCR performance. Mol. Asp. Med..

[60] Xu J.T., Zhao X., Yaster M., Tao Y.X. (2010). Expression and distribution of mTOR, p70S6K, 4E-BP1, and their phosphorylated counterparts in rat dorsal root ganglion and spinal cord dorsal horn. Brain Res..

[61] Hayat M.A. (1981). Principles and Techniques of Electron Microscopy. Biological Applications.

[62] Wiame I., Remy S., Swennen R., Sagi L. (2000). Irreversible heat inactivation of DNase I without RNA degradation. BioTechniques.

